# Enhancing PET/CT Radiomics Robustness Through Graph Signal Processing

**DOI:** 10.3390/diagnostics16142284

**Published:** 2026-07-21

**Authors:** Tommaso Latino, Alessandro Stefano, Giovanni Pasini, Franco Marinozzi, Giorgio Russo, Fabiano Bini

**Affiliations:** 1 Department of Mechanical and Aerospace Engineering, Sapienza University of Rome, Eudossiana 18, 00184 Rome, Italy; latino.1903488@studenti.uniroma1.it (T.L.); franco.marinozzi@uniroma1.it (F.M.); fabiano.bini@uniroma1.it (F.B.); 2 Institute of Bioimaging and Complex Biological Systems, National Research Council, 90015 Cefalù, Italy; giovannipasini@cnr.it (G.P.); giorgio-russo@cnr.it (G.R.); 3 National Laboratory of South, National Institute for Nuclear Physics (LNS-INFN), 95123 Catania, Italy

**Keywords:** radiomics, graph-based feature extraction, graph signal processing, spectral analysis, machine learning

## Abstract

**Background/Objectives**: Prostate cancer (PCa) frequently metastasizes to bone, leading to severe clinical complications and reduced quality of life. Accurate and robust imaging-based characterization of bone lesions is therefore critical for diagnosis and treatment planning. Radiomics has emerged as a powerful tool for extracting quantitative information from medical images; however, classical radiomics features are often affected by inter-scanner variability, segmentation dependence, and limited ability to describe lesions with complex biological heterogeneity. This study aims to introduce a translational graph-based radiomics approach designed to extract novel quantitative descriptors with improved robustness and clinical reliability. **Methods**: A graph representation was derived from segmented Positron Emission Tomography/Computed Tomography (PET/CT) bone lesions by generating a point cloud followed by Delaunay triangulation to preserve geometric information. Graph signal processing techniques were applied to extract three classes of features: orientation, connectivity, and transform-based descriptors. The dataset included PET/CT scans from 50 PCa patients acquired using two different scanners, comprising 92 bone lesions classified as benign or malignant. Correlation analysis with classical radiomics features was performed to assess information redundancy. Robustness against batch effects and segmentation variability was evaluated. Classification performance was tested using Linear Discriminant Analysis (LDA) and Support Vector Machine (SVM) models based on proposed features, classical features, and their combination. **Results**: The proposed features captured non-redundant information compared to classical radiomics and demonstrated superior robustness to scanner-related batch effects and segmentation variability. In classification tasks, models using the proposed features consistently outperformed those based on classical radiomics. Using LDA, the proposed features achieved a mean balanced accuracy of 69.68% and a mean Area Under the Curve (AUC) of 72.14%. With SVM, they achieved a mean balanced accuracy of 65.16% and a mean AUC of 66.49%, exceeding the performance of classical and combined feature sets. **Conclusions**: This study presents a translational graph-based radiomics framework that extends beyond conventional methodologies, improving robustness and diagnostic performance. The proposed approach shows promise as an integrative tool for more reliable PET/CT-based characterization of bone lesions in prostate cancer.

## 1. Introduction

Prostate cancer (PCa) is one of the leading causes of cancer-related death among men. In autopsy studies, bone metastases are present in approximately 90.1% of men who died from metastatic prostate cancer [1,2,3]. Tumour cells detach from the primary tumour through epithelial-to-mesenchymal transition (EMT) and prepare optimal proliferative conditions through exosome release. After prior intravasation, tumour cells reach bone tissue and begin forming metastases through either the destruction (osteolytic metastases) or disorganized formation (osteoblastic metastases) of bone tissue, causing complications that may lead to physical, metabolic, and neurological effects [4]. For adequate prevention, it is essential to have an accurate diagnosis to identify an appropriate interventional or therapeutic pathway. However, it is not uncommon for a patient’s clinical profile, due to factors related to complex etiology or possible intra-subject heterogeneity, to fall within a clinical context where confounders are numerous and the discriminative elements between physiological and pathological conditions are limited or not easily identifiable through visual inspection of images derived from conventional diagnostic examinations [5]. In this complex scenario, radiomics has emerged as a computational imaging approach that has taken on a leading role in clinical research, owing to its effectiveness in providing concrete support tools from both diagnostic and therapeutic perspectives [6,7,8,9,10,11]. Radiomics is based on a set of procedures that allow the extraction of quantitative information or “features” from medical images [12,13], which are used for predictive modelling [10,14,15], with the aim of discriminating the clinical condition of a patient. A standard radiomics pipeline includes preprocessing, segmentation of the volume of interest [16,17], feature extraction [18], and model development using machine learning algorithms [19]. However, the workflow of a standard radiomics analysis is characterized by intrinsic limitations [20,21]. Scanner characteristics, including hardware and acquisition parameters, define the voxel grid on which images are reconstructed, while segmentation determines which voxels are included in the analysis. Therefore, differences in scanner hardware and acquisition settings, together with the inter-/intra-operator variability introduced by manual segmentation, directly impact radiomics features, as they determine the voxel grid that defines the data and the intensity scales associated with each voxel. Although research efforts have aimed to automate segmentation and statistical methods such as ComBat [22,23] have been proposed to mitigate scanner-induced variability, none of these approaches fully resolve the intrinsic dependence of radiomics features on acquisition, segmentation, and voxel discretization. It is also common for radiomics features to fail to capture the complex morpho-functional patterns underlying specific clinical conditions, limiting their ability to fully represent the lesions’ phenotype [24,25]. The construction of robust models is therefore often precluded by the intrinsic nature of the input data. Although radiomics originated as a stand-alone technique characterized by standard procedures in the literature, the data and objects processed during the analysis are sufficiently versatile to enable the integration of approaches and results suitable for completely different scientific and operational fields. This study introduces the concept of point clouds into radiomics analysis. A point cloud is an unstructured set of points that may derive from or represent volumes and specific spatial structures [26]. The connection with the underlying structure is latent but can be made explicit by applying appropriate processing techniques. This scenario allows, with appropriate precautions, the mapping of data into a completely different analytical domain compared to that in which the target volume is defined, reducing the dependence of the representation on the original grid. To make the new representation robust and informative, we applied a strategy to compensate for the controlled loss of information caused by extracting the point clouds from the volumes. The solution we adopted to recover the underlying geometric information consisted of using the extracted point clouds as input to construct meshes [27] that accurately approximated the volumes, allowing us to integrate graph signal processing (GSP) [28] tools for feature extraction. In this study, 50 patients, for a total of 92 lesions, were selected to evaluate the pipeline. For each patient, standard radiomics features were extracted in accordance with the Image Biomarker Standardisation Initiative (IBSI) [29], along with the features derived from the proposed analysis. The study was structured around three primary objectives: (i) to validate the additional information content of the proposed features; (ii) to verify the robustness of GSP predictors to variability introduced by segmentation and batch effects; and (iii) to assess their ability to build robust predictive models, either as stand-alone inputs or in combination with classical features. For the first objective, a patient-level Spearman correlation analysis was performed [30]. For the second objective, classical and GSP features were recomputed after introducing perturbations to lesion segmentation masks, and percentage differences were analysed using descriptive statistics to evaluate sensitivity to segmentation variability. Batch effects were further investigated at the feature level by stratifying patients according to scanner type and testing group differences using the Kruskal–Wallis test [31]. For the third objective, the classification performance was estimated primarily using Receiver Operating Characteristic (ROC) analysis and balanced accuracy. A comprehensive panel of additional classification metrics was also evaluated to provide a complete performance profile. The Results section reports the outcomes of the analyses and classification performance, and the Discussion addresses the strengths and limitations of the proposed framework.

## 2. Materials and Methods

**Figure 1:**
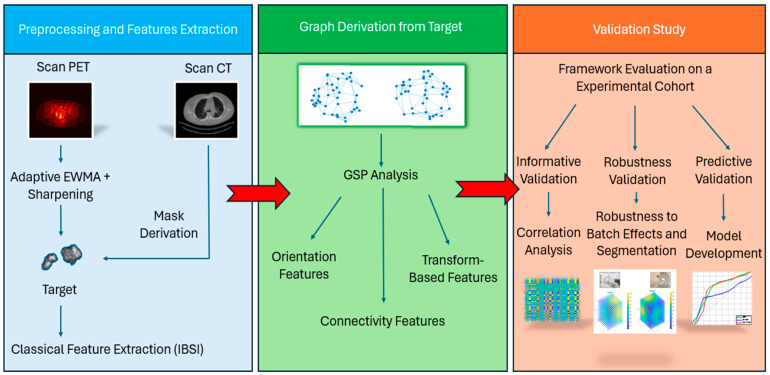
Framework flowchart. The PET/CT images are processed using an adaptive EWMA and a sharpening filter, and then classical IBSI-compliant radiomics features are extracted from the segmented volume. A graph is subsequently derived from the lesion, and graph signal processing tools are used to extract three distinct types of descriptors. The framework was tested on a cohort to assess information redundancy with classical predictors, feature robustness against batch effects and segmentation variability, and machine learning classification performance.

In this study, the PET/CT images were pre-processed through an adaptive EWMA and sharpening filtering chain. Each lesion was segmented using masks derived from computed tomography (CT) scans. From the resulting segmented volume, IBSI-compliant radiomics features [29] were extracted from the corresponding positron emission tomography (PET) images. Subsequently, a point cloud was extracted from the volume, the mesh was constructed, and GSP analysis was applied to compute the proposed features, which include three distinct types of descriptors: orientation, connectivity, and transform-based features. From each lesion, 206 classical features (comprising 49 intensity, 21 shape, and 136 texture features) and 77 GSP features were extracted. At the end of the analysis, the two sets of features and the combined one were used to perform a patient-level correlation analysis, to verify the robustness of GSP predictors to the variability introduced by segmentation and to batch effects, and to train a Linear Discriminant Analysis (LDA) classifier and a Support Vector Machine (SVM) classifier. For each classifier, three different models were trained, and their predictive performance was evaluated. To provide a comprehensive overview of the proposed framework, a detailed flowchart outlining the sequential steps of image preprocessing, classical feature extraction, graph-based processing, and the subsequent validation study is illustrated in Figure 1.

### 2.1. The Dataset

The dataset consisted of PET/CT images acquired from 50 patients, comprising a total of 92 lesions, with two different scanners and stored in DICOM format. For each patient, an average of 290 slices was acquired. Each lesion was associated with an identifying label, resulting in a total of 75 benign bone lesions and 17 malignant lesions.

### 2.2. Preprocessing

The entire pipeline is designed to operate on PET images, which are inherently noisy by construction and characterized by very low spatial resolution due to the technological limitations of scanner components and low-count phenomena. To correctly extract the diagnostic content from the images, we developed a specific filtering pipeline aimed at mitigating the intrinsic issues of PET data.

An adaptive Exponentially Weighted Moving Average (EWMA) filter was implemented to improve the local Signal-to-Noise Ratio (SNR). The filter output is then convolved with a sharpening kernel. Since the targets of interest generally stand out from the surrounding tissue, the filtering pipeline is designed to emphasize these transitions while simultaneously reducing residual noise.

#### 2.2.1. The Adaptive EWMA Filter

The EWMA filter is a particular variant of the classic moving average widely used in numerical signal analysis. The main issue with the standard moving average, which makes it unsuitable in this context, concerns the inability to robustly control the amount of smoothing. This could seriously compromise the result, with a high risk of introducing excessive blurring and thereby eliminating diagnostic information. The EWMA variant partially addresses this problem thanks to the possibility of actively adjusting the amount of smoothing applied during filtering. Let us consider the one-dimensional sequence $x_{n}$ and assume it is passed through an EWMA filter. The output $y_{n}$ will have the following form:

(1) $$y_{n}=\alpha x_{n}+\left(1-\alpha \right)y_{n-1},\;0<\alpha <1$$

The smoothing action is controlled through the parameter α. A low weight factor emphasizes the contribution of the previous output, reducing the influence of the current input, while a high weight factor, conversely, emphasizes the current input and reduces the contribution of the previous output. Low weight factors therefore correspond to very aggressive filtering, whereas high values favour fidelity to the input sequence, resulting in much lighter smoothing. Based on the one-dimensional formulation, we developed a two-dimensional extension of the filter in the image domain. Let $x[n_{1},n_{2}]$ be the input image; the output $y[n_{1},n_{2}]$ will take the following form:

(2) $$\left\{\begin{array}{c} y^{\left(h\right)}\left[n_{1},n_{2}\right]=\left(1-\alpha \right)\;y^{\left(h\right)}\left[n_{1},n_{2}-1\right]+\alpha \;x\left[n_{1},n_{2}\right]\\ y\left[n_{1},n_{2}\right]=\left(1-\alpha \right)\;y\left[n_{1}-1,n_{2}\right]+\alpha \;y^{\left(h\right)}\left[n_{1},n_{2}\right] \end{array}\right.\;\;,\;\;0<\alpha <1$$

Filtering is performed in two steps: first, a row-wise filtering is performed producing the temporary output $y^{\left(h\right)}[n_{1},n_{2}]$; subsequently, this is used as input for column-wise filtering, producing the final output $y[n_{1},n_{2}]$. Although the EWMA filter in this form allows global filtering control, it does not allow control or modulation of local smoothing. This could be critical, especially in clinical contexts where local gradients are usually high due to anatomical complexity. Therefore, we developed an adaptive EWMA filter, capable of actively modulating the smoothing parameter based on the local image gradient. The idea is to apply strong smoothing in flat, low-gradient areas (usually less informative), and almost no smoothing in high-gradient areas rich in details, such as edges and corners. This approach improves local SNR while preserving high-spatial-frequency structures and avoiding blurring the target boundaries to be segmented. To estimate the image gradient, Sobel filters were used: they are two Finite Impulse Response (FIR) kernels that allow gradient estimation. Their matrix definitions are:

(3) $$S_{\mathrm{row}}\left[n_{1},n_{2}\right]=\left[\begin{array}{ccc} -1 & 0 & 1\\ -2 & 0 & 2\\ -1 & 0 & 1 \end{array}\right]$$

(4) $$S_{\mathrm{column}}\left[n_{1},n_{2}\right]=\left[\begin{array}{ccc} -1 & -2 & -1\\ 0 & 0 & 0\\ 1 & 2 & 1 \end{array}\right]$$

Convolving the original image with $S_{row}$ and $S_{column}$ estimates the gradient components along rows and columns, respectively. Let $G_{x}[n_{1},n_{2}]$ be the row component and $G_{y}[n_{1},n_{2}]\;$ the column component. The gradient is computed as:

(5) $$\;G\left[n_{1},n_{2}\right]=\sqrt{G_{x}^{2}\left[n_{1},n_{2}\right]+G_{y}^{2}\left[n_{1},n_{2}\right]}$$

The gradient is then normalized to [0, 1] to make smoothing control scale-invariant. The modulation of the weight parameter α is performed using a Gaussian kernel applied to the normalized gradient:

(6) $$\alpha \left[n_{1},n_{2}\right]=\alpha_{min}+\left(\alpha_{max}-\alpha_{min}\right)\left(1-\exp\left(-\frac{G_{\mathrm{norm}}^{2}\left[n_{1},n_{2}\right]}{2\sigma^{2}}\right)\right)$$

This relationship describes the behaviour discussed above. The parameter σ determines the weight parameter’s sensitivity to the gradient, while $\alpha_{min}$ and $\alpha_{max}$ indicate the minimum and maximum weight values, corresponding to the maximum and minimum smoothing. The parameters used in the framework are shown in Table 1. The optimisation strategy for this parameter set was strictly driven by the need to find a rigorous trade-off between noise reduction (image quality) and spatial detail preservation (mitigation of blurring).

**Figure 2:**
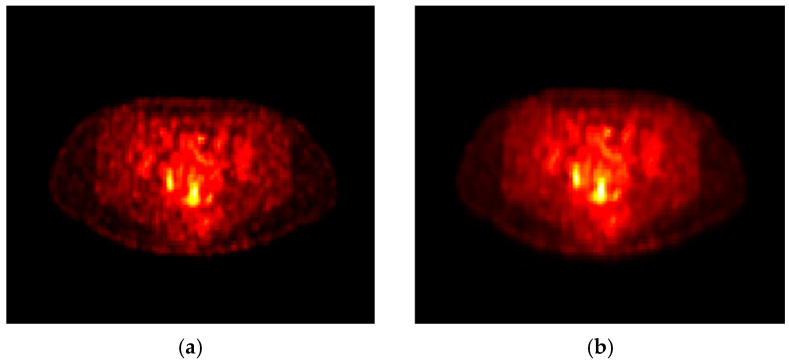
Adaptive EWMA filter output: (**a**) Original PET slice; (**b**) Filtered PET slice. The parameter configuration adopted improves the local SNR while controlling excessive blurring and preserving diagnostic details.

In Figure 2, a representative PET slice from the dataset is shown (a), together with the corresponding output of the adaptive EWMA filter (b).

#### 2.2.2. The Sharpening Filter

The second filtering block is a sharpening kernel. It is a FIR filter that extracts the high-spatial-frequency components of the image (e.g., edges, corners) and adds them to the input image, which in this case is the output of the adaptive EWMA filter. The result coincides with the original image enriched with details. We adopted a 3 × 3 sharpening kernel to avoid excessively amplifying residual noise, which would negate the EWMA effect. A basic 3 × 3 kernel has the following form:

(7) $$\mathrm{Sharp}\left[n_{1},n_{2}\right]=\left[\begin{array}{ccc} 0 & -1 & 0\\ -1 & 5 & -1\\ 0 & -1 & 0 \end{array}\right]$$

**Figure 3:**
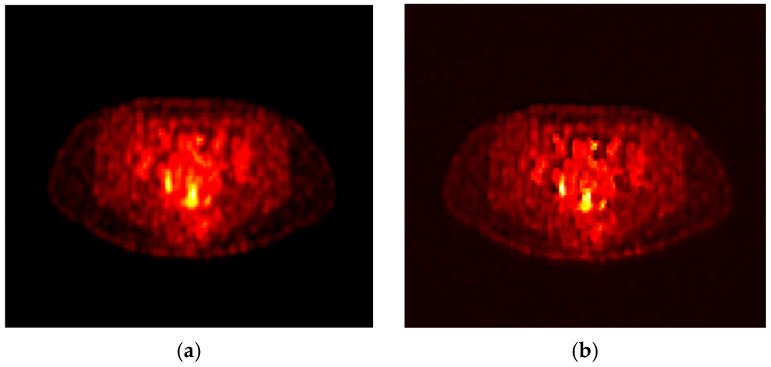
Filtering chain output: (**a**) Original PET slice; (**b**) Filtered PET slice. The filtering pipeline emphasizes high-frequency details while simultaneously reducing residual noise.

Let us consider the matrices:

(8) $$\delta \left[n_{1},n_{2}\right]=\left[\begin{array}{ccc} 0 & 0 & 0\\ 0 & 1 & 0\\ 0 & 0 & 0 \end{array}\right]$$

(9) $$\mathrm{Lap}\left[n_{1},n_{2}\right]=\left[\begin{array}{ccc} 0 & -1 & 0\\ -1 & 4 & -1\\ 0 & -1 & 0 \end{array}\right]$$

where $\delta [n_{1},n_{2}]$ represents the spatial impulse and $Lap[n_{1},n_{2}]$ represents a Laplacian kernel. The latter is a high-pass filter that extracts high-spatial-frequency components by subtracting the average of neighbouring elements from the current element. If the area is flat, the filter output is zero, whereas if the current element stands out from its context, the filter detects it. Comparing the 7 with the 8 and 9, the sharpening kernel can be rewritten as the sum of the spatial impulse and the Laplacian kernel. Let $x[n_{1},n_{2}]$ be the input image, then the output $y[n_{1},n_{2}]$ can be written as:

(10) $$y\left[n_{1},n_{2}\right]=x\left[n_{1},n_{2}\right]*\left(\delta \left[n_{1},n_{2}\right]+\mathrm{Lap}\left[n_{1},n_{2}\right]\right)=x\left[n_{1},n_{2}\right]+x_{H}\left[n_{1},n_{2}\right]$$

where $x_{H}[n_{1},n_{2}]$ is the high-spatial-frequency component of the input image. Figure 3 shows the original slice (a) and the output of the complete filtering chain (b), where the adaptive EWMA output in Figure 2b was convolved with the sharpening kernel.

### 2.3. Standard Radiomics Features

Classical radiomic features are quantitative descriptors used to characterize specific properties of segmented volumes. According to the IBSI guidelines [29] three main classes of standardized descriptors are defined:

• **Intensity features**: This category includes all first-order statistical metrics that can be extracted from the grey-level distribution of the segmented volume.
• **Shape features**: This class of metrics describes the geometric characteristics of the target.
• **Texture features**: This category of features includes metrics derivable from the spatial distribution of specific patterns associated with voxel intensity.

### 2.4. Segmentation and Classical Feature Extraction

The pipeline was applied to PET images, as these provided information for the functional patterns of interest, whereas CT was used for anatomical reference [32,33] to create the masks delineating the lesions. For feature extraction, the Medical Imaging Toolbox of MATLAB 2023b [34] was employed; specifically, upon loading and filtering the images, the metadata were extracted and combined with the volumetric data to create a radiomics object, from which the standard features were extracted using the toolbox’s built-in functions with default parameters.

### 2.5. Point Cloud Extraction and Mesh Construction

**Figure 4:**
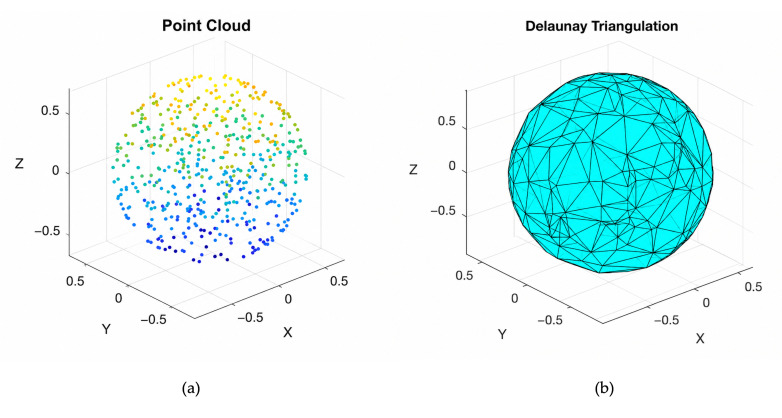
Toy example: (**a**) Spherical point cloud; lighter colors for positive z and darker colors for negative z (**b**) Delaunay triangulation. The algorithm connects the input points into robust, well-conditioned tetrahedra ensuring that no point lies inside their circumscribed spheres. This geometric property allows accurate curvature preservation while reducing grid artifact dependencies during the volume-to-mesh transformation.

From the segmented volume, a pseudorandom subset of voxels was extracted while strictly controlling the size of the point cloud. Through preliminary experiments, a maximum size of 600 points was determined as the optimal trade-off between the quality of the geometric representation and computational time (for more details, see Section S1 in the Supplementary File). Automatic subsampling was applied to point clouds exceeding this limit, reducing the number of points to 600. Once the point cloud was extracted, we applied the Delaunay algorithm [35] to construct the mesh. It is a widely used algorithm in computer vision due to its ability to build robust and reliable meshes. Delaunay triangulation takes the points as input and connects them with tetrahedra such that no point of the point cloud lies inside the circumscribed sphere of any tetrahedron. This property allows the construction of regular and well-conditioned tetrahedra, avoiding distortion of the local geometric curvature while reducing dependence on the regular grid on which the target volume is defined. As a toy case, Figure 4 shows an example of a spherical point cloud (a) and its corresponding Delaunay triangulation (b).

#### 2.5.1. Orientation Features

Let us consider only the external triangles of a Delaunay triangulation obtained by considering only the extreme points from the original point cloud. Let $T\left(v_{1},v_{2},v_{3}\right)$ denote a generic triangle of the mesh defined by vertices $v_{1},v_{2},v_{3}$. Let vectors $a,b\in R^{3}$ be defined as:

(11) $$a=v_{2}-v_{1},\;b=v_{3}-v_{1}$$

We define now the unit normal:

(12) $$\hat{n}=\frac{a\times b}{\left\|a\times b\right\|}$$

Let θ(in degrees) be the angle between $\hat{n}$ and the mean normal $\hat{n_{mean}}$:

(13) $$\theta =\arccos\left(\hat{n}\cdot \hat{n_{\mathrm{mean}}}\right)\cdot \frac{180}{\pi }$$

From the Delaunay triangulation, the boundary triangles were identified, and, for each triangle, the normal vector was computed together with its angular dispersion relative to the mean normal vector. From the multivariate distribution built from the coordinates of all triangle normals, univariate statistics such as mean, median, percentile-based descriptors, maximum, minimum, variance, skewness, and kurtosis were computed for each coordinate distribution. The same analysis was applied to the angular distribution. These statistical descriptors, particularly dispersion and shape metrics, provided complementary measures of global surface orientation and geometric irregularity. To identify the presence of preferential directions, the statistical entropy of the angular distribution was computed according to the following formulation:

(14) $$H=-\sum_{i}p\left(i\right)\;\log p\left(i\right)$$

where $p\left(i\right)$ represents the probability that an observation falls into the i-th bin of the normalized histogram. A high entropy indicates the absence of preferential orientations, suggesting a more isotropic surface configuration, whereas lower entropy values reflect the presence of dominant directional patterns.

#### 2.5.2. Connectivity Features

Let us consider the complete mesh, including internal triangle connections. We can now define $G=\left(V,E\right)$ as the graph associated with the point cloud, where V denotes the set of points and E the set of connections (edges) between them. The adjacency matrix A is defined as the binary matrix that describes the connections between nodes [36]. Each element of the matrix takes the value 1 if the corresponding pair of nodes is connected, 0 otherwise:

(15) $$A_{ij}=\left\{\begin{array}{c} 1\;if\;\;\left(v_{i},v_{j}\right)\in E\\ 0\;otherwise \end{array}\right.$$

Considering the generic node $v_{i}$, we can define its degree as the number of nodes to which it is directly connected [28]:

(16) $$d_{i}=\sum_{j=1}^{N}A_{ij}$$

where $d_{i}$ denotes the degree and N the number of nodes. From this last definition, it is possible to introduce the Laplacian operator [36] as the difference between the diagonal matrix containing the degrees of all nodes composing the graph, and the adjacency matrix. Let us consider the endomorphism reported below:

(17) Lv=λv

where λ and v denote respectively the generic eigenvalue and eigenvector of the Laplacian. From the eigenvalue and eigenvector decomposition it is possible to infer some interesting properties of the graph such as connectivity and its topological complexity [28]. The eigenvectors correspond to the basis for signals associated with the nodes of the graph; they indicate the modes of variation supported by the topology. The eigenvalues, instead, encode the spatial frequencies associated with the eigenvectors. Eigenvectors with strong nodal variability will be associated with high eigenvalues and vice versa. The first eigenvalue is always equal to 0, which corresponds to the constant eigenvector and is always supported by all nodes [36]. From the Delaunay mesh, we computed the graph Laplacian and its eigenvalue–eigenvector decomposition. We used the extreme-position statistical metrics on the distribution of eigenvalues (excluding the null eigenvalue) to estimate the topological complexity of the graph, while the dispersion and orientation metrics provided estimates of the average density of connections and possible inhomogeneities.

#### 2.5.3. Features Derived from Transforms

From the mesh representation, it is possible to define a nodal signal associated with the graph. From a radiomics perspective, since the point cloud underlying the graph is a subset of the voxels composing the segmented volume, we assigned each point the grey level (radiotracer activity) it had in the original image. Within the graph analysis, we then applied transformations that allowed the extraction of information regarding the radiotracer activity while accounting for the topological structure on which the signal was defined.

##### Features Derived from the Graph Fourier Transform

**Figure 5:**
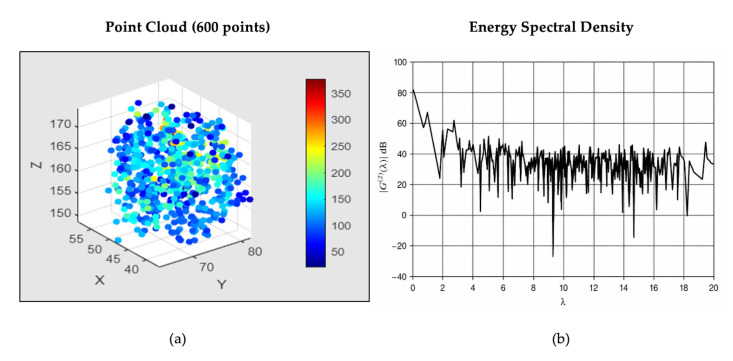
Graph spectral decomposition: (**a**) Signal on graph; (**b**) Energy spectral density. The spectral coefficients quantify the similarity between the lesion spatial signal and the graph Laplacian eigenvectors, capturing the structural modes of variation supported by the topology.

The Graph Fourier Transform (GFT) [37] is the graph equivalent of the classical Fourier transform. The bases, in this case, are not sinusoids but the eigenvectors of the Laplacian. Since “transforming” a signal means projecting it onto a new basis where each new element is calculated by performing a similarity measure (correlation) between the signal and the generic basis element, the GFT assumes the following synthesis formulation:

(18) $$G=\Phi^{⊤}f$$

where G denotes the transform, f is the node signal and Φ is the matrix of Laplacian eigenvectors. The GFT is characterized by both frequency and spatial localization. The coefficients establish the similarity with the modes of variation supported by the graph. Now consider the energy spectral density [37] obtained from G. The i-th coefficient can be computed according to the following formulation:

(19) $$\mathrm{ES}D_{i}={\left|G_{i}\right|}^{2},i=1,\ldots ,N$$

where $G_{i}$ represents the i-th spectral coefficient. Figure 5 shows an example of signal on graph extracted from a lesion in the dataset (a) and the corresponding energy spectral density (b). For greater graphical clarity, the connections between the nodes have not been shown.

From this representation, we extracted features that quantified the total energy and its distribution. The energy was normalized by the total number of nodes to compare the total metabolic activity across graphs of different sizes:

(20) $$E=\frac{1}{N}\sum_{i=1}^{N}\mathrm{ES}D_{i}$$

For the computation of the metrics, it was preferable to filter out the constant component (first eigenvalue). We separated low frequencies from high frequencies [38] by defining a threshold value $\lambda_{th}$:

(21) $$L=\{\;i|\lambda {\;⠀}_{i}\leq \lambda_{\mathrm{th}}\;\}$$

(22) $$H=\{\;i|\lambda_{i}>\lambda_{\mathrm{th}}\;\}$$

We evaluated the energies associated with the two frequency bands by summing the spectral coefficients corresponding to the bands of interest:

(23) $$E_{\mathrm{LF}}=\frac{1}{N}\sum_{i\in L}\mathrm{ES}D_{i}$$

(24) $$E_{\mathrm{HF}}=\frac{1}{N}\sum_{i\in H}\mathrm{ES}D_{i}$$

**Figure 6:**
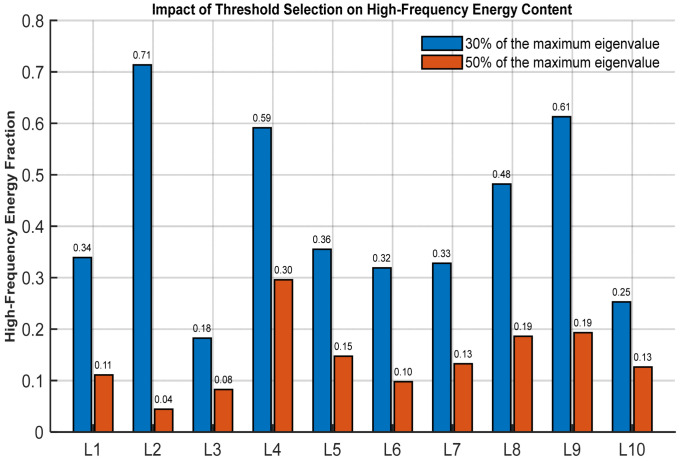
Impact of threshold on frequency decomposition. The 50% threshold restricts the high-frequency contribution, whereas the 30% threshold ensures a balanced spectral decomposition where both global trends and local signal fluctuations are represented.

By normalizing these two quantities by the total energy, we obtained a relative measure of energy partition between the two bands. To separate the frequency range of the energy density spectrum derived from the GFT, we used a threshold equal to 30% of the maximum eigenvalue. To show the rationale behind this operational approach, 10 lesions from different patients in the dataset were considered. For each lesion, the high-frequency energy component was calculated, selecting the spectral threshold at 30% and 50% of the maximum eigenvalue, respectively. The results of the procedure are reported in Figure 6.

From the figure, the threshold greatly influences the results. In this scenario, the choice of a threshold equal to 50% of the maximum eigenvalue tends to concentrate most of the energy in the low-frequency band, reducing the contribution of high-frequency components and limiting the sensitivity to localized variations, which are often indicators of potential metabolic peaks in the signal. A threshold equal to 30% allows a balanced spectral decomposition, where both global trends and local fluctuations of the signal are meaningfully represented.

##### Features Derived from the Graph Wavelet Transform

**Figure 7:**
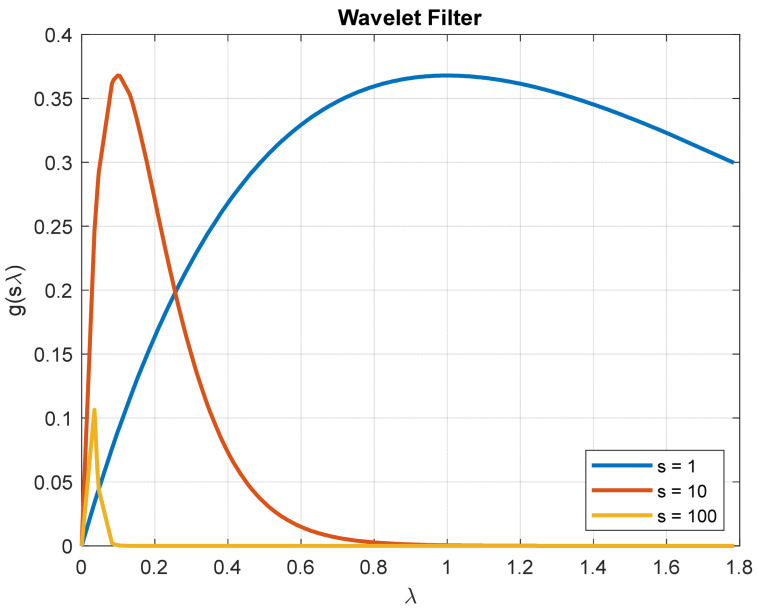
Wavelet kernel at different scales. The frequency response highlights the transition from the high-frequency regime at s = 1 (broadband response for localized variations) through the intermediate-frequency regime at s = 10, up to the low-frequency regime at s = 100 (narrowband response for macroscale patterns), ensuring a seamless and comprehensive spectral coverage.

The second type of transform we considered was the Graph Wavelet Transform (GWT) [39]. This approach allows for investigating how the nodal signal varies with respect to its context across different spatial scales. For the analysis, we defined weighting factors for the graph before applying the decomposition. We built weighting factors that reflected the Euclidean distances between connected nodes, modulating them through a Gaussian kernel [39]:

(25) $$W_{ij}=\left\{\begin{array}{c} \exp\left(-\frac{|x_{i}-x_{j}{|}_{2}^{2}}{2\sigma^{2}}\right)\\ 0\;otherwise \end{array}\;\right.$$

where $W_{ij}$ denotes the weight assigned to the edge between node i and node j. If the edge does not exist, no weight is assigned. Then, we introduced the matrix D, a diagonal matrix where the generic diagonal element is the sum of all weights associated with the corresponding node and we used it to build the normalized Laplacian [36,39]:

(26) $$L=I-D^{-1/2}WD^{-1/2}$$

where I is the identity matrix and W is the weight matrix. We used the normalized Laplacian for the GWT to enable a more interpretable multi-scale analysis, as normalization mitigates the influence of varying node degrees and weighted edges, producing spectral components that are stable and comparable across nodes and scales. As in the classical wavelet transform, it was necessary to identify a wavelet kernel to use for the decomposition. Following Hammond et al., we adopted one of the proposed spectral kernels, defined as:

(27) $$g\left(s\lambda \right)=s\lambda \;e^{-s\lambda }$$

where λ is an eigenvalue of the normalized Laplacian and s represents a specific scale. It is a band-pass filter that emphasizes specific frequency bands. Small scales correspond to broader bands and a theoretical peak shifted toward higher frequencies, allowing the capture of local variations that manifest over a relatively extended frequency support. Large scales correspond to narrower bands and a theoretical peak shifted toward lower frequencies, enabling the filter to selectively highlight patterns that manifest at a macroscale. To apply the wavelet, we selected three different spatial scales: s=1, s=10, and s=100. The choice of wavelet scales was guided by the spectral distribution of the graph Laplacian kernel, to sample representative low-, intermediate-, and high-frequency regimes of the graph spectrum. Figure 7 shows the shape of the kernel for the chosen scales.

**Figure 8:**
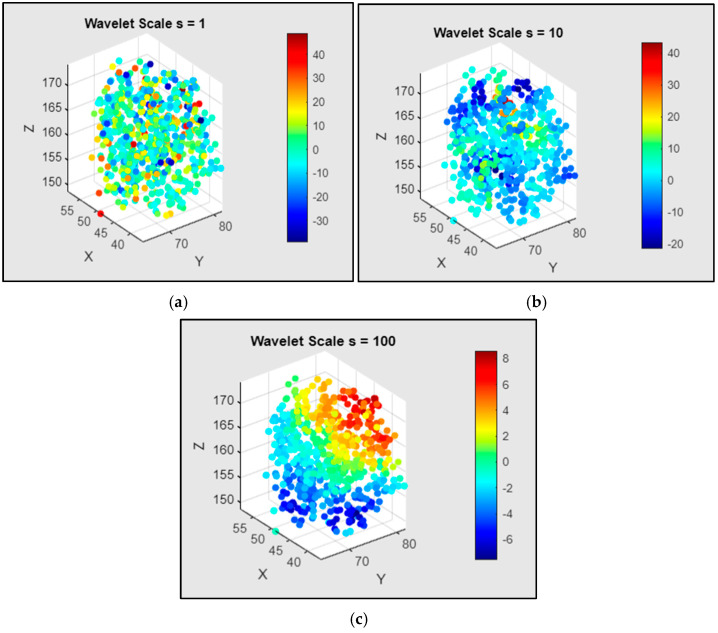
Graph Wavelet Transform: (**a**) s=1; (**b**) s=10; (**c**) s = 100. The plots track the spatial variation of the transform output, where positive and negative coefficient amplitudes denote localized signal variations relative to the scale-dependent context. The transition from (**a**) to (**c**) demonstrates the multi-resolution filtering operation.

To apply the GWT, the node signal must be projected onto the eigenvectors of the normalized Laplacian [39]. The GFT is subsequently weighted by the kernel. Finally, the result is reprojected onto the nodal space through the eigenvector matrix:

(28) $$\psi {\;⠀}_{s}=U\;g\left(s\lambda \right)\;U^{⊤}f$$

where $\psi_{s}$ denotes the wavelet transform, U the eigenvector matrix, and f the nodal signal. Positive nodal coefficients reflect an increase in the nodal signal with respect to the local context determined by the scale, whereas negative nodal coefficients reflect a decrease. For each scale, the decomposition was performed, resulting in three distinct distributions representing the radiotracer activity on the graph at the selected spatial resolutions. From the wavelet distributions, we used the statistical metrics listed in Section 2.5.1 to extract information on trend and dispersion patterns at different scales. Figure 8 shows an example of wavelet decomposition applied to the signal in Figure 5a.

### 2.6. Validation Analysis

The procedures described above were repeated for all lesions, and at the end of the process, the resulting data were used to assess the reliability of the proposed methodology through informative, robustness, and predictive validation.

### 2.7. Informative Validation

To evaluate the added informational value of the new features, a correlation analysis was conducted using Spearman’s correlation. From the correlation matrix, the distribution of the average correlation with the classical features was subsequently derived for each proposed feature, and a left-tailed Wilcoxon signed-rank test [40] was performed to test the median of the distribution against different reference values.

### 2.8. Robustness Validation

The dependence on segmentation was evaluated by recomputing the classical and GSP metrics extracted from a subset of 10 lesions after perturbing the original mask used for target extraction. The percentage differences between the classical and GSP feature vectors were analysed using a descriptive statistical approach to assess robustness to the variability introduced by mask perturbation. The dependence of classical and GSP features on batch effects was evaluated at the patient level. For each feature, the distribution of its observations was considered. This distribution was split into two groups based on scanner membership, and the Kruskal–Wallis test was performed to assess the presence of batch effects. Given the high number of comparisons to assess the presence of batch effects, and to control for false positives, the *p*-value of each comparison was corrected (*q*-value) using the Benjamini–Hochberg False Discovery Rate (FDR) correction [41]:

(29) $$q_{\left(i\right)}=\frac{p_{\left(i\right)}\cdot n}{i}$$

where $p_{\left(i\right)}$ represents the *p*-value corresponding to the i-th comparison, n the number of tests, and i the rank that the *p*-value assumes in the distribution composed of the *p*-values of the various comparisons.

### 2.9. Predictive Validation

To assess the discriminative power of the proposed features, we implemented two different Machine Learning methods: LDA [42] and SVM [43]. These models were selected because the dataset is relatively small, high-dimensional, and affected by class imbalance. In this context, linear models are preferred due to their lower variance and reduced risk of overfitting compared to more complex nonlinear approaches. In particular, the diagonal covariance assumption in LDA was adopted to improve numerical stability in small-sample settings, where full covariance estimation can be unreliable. Similarly, a linear kernel SVM was chosen to maintain model simplicity, avoid additional hyperparameter complexity associated with nonlinear kernels, and ensure robustness in low-sample regimes. For each algorithm, three models were constructed and trained on the proposed features, the classical features, and a combination of both, respectively. The classification pipeline was implemented using a repeated, patient-wise stratified 5-fold cross-validation scheme. Specifically, patients were used as the unit of splitting to avoid any information leakage between training and testing sets, ensuring that all observations from the same patient were assigned to the same fold. A stratified grouping strategy was adopted to preserve the proportion of classes across folds. Within each training fold, feature selection was performed independently using Least Absolute Shrinkage and Selection Operator (LASSO) [42], applied exclusively to the training data. The top-ranked features were then selected and used for model training. This procedure was repeated separately within each cross-validation split, ensuring that no information from the test set was used during feature selection and thereby preventing data leakage. To address class imbalance, a misclassification penalty (class weighting) was introduced during training. The weight assigned to the minority class was set proportional to the inverse class frequency in the training set, thereby encouraging a more balanced contribution of both classes to the decision boundary. Performance was evaluated on unseen test folds and aggregated across 10 repetitions of the entire cross-validation procedure to reduce variance in the estimates.

#### 2.9.1. Performance Metrics

The predictive performance of a model can be quantified using several metrics. Considering a positive-negative dichotomy, the most relevant metrics are:

• **True Positive (TP)**: Total positive observations correctly classified. Normalized by the total number of positives, this gives the True Positive Rate (TPR), also called sensitivity or recall.
• **True Negative (TN)**: Total negative observations correctly classified. Normalized by the total number of negatives, this gives the True Negative Rate (TNR), also called specificity.
• **False Positive (FP)**: Total negative observations incorrectly classified as positive. Normalized by the total number of negatives, this gives the False Positive Rate (FPR), also called fall-out.
• **False Negative (FN)**: Total positive observations incorrectly classified as negative. Normalized by the total number of positives, this gives the False Negative Rate (FNR).

These metrics can be combined to estimate the accuracy, which represents the overall discriminative ability of the model. It is defined as the ratio of correctly classified observations to the total number of observations:

(30) $$Accuracy=\frac{TP+TN}{TP+TN+FP+FN}$$

In the presence of class imbalance, Balanced Accuracy is preferred to prevent an overoptimistic performance evaluation. It is calculated as the arithmetic mean of sensitivity and specificity:

(31) $$Balanced\;Accuracy=\frac{Recall+Specificity}{2}$$

To gain a deeper insight into the model’s performance, especially regarding the positive class, Precision and F1-score are also evaluated. Precision quantifies the model’s reliability when predicting the positive class and is defined as the ratio of correctly classified positive observations to the total predicted positives:

(32) $$Precision=\frac{TP}{TP+FP}$$

The F1-score is calculated as the harmonic mean of precision and recall. It provides a single, balanced metric that accounts for both false positives and false negatives, proving particularly useful when dealing with uneven class distributions:

(33) $$F1\text{-}score=2\cdot \frac{Precision\cdot Recall}{Precision+Recall}$$

Class assignment is determined by comparing the score to a threshold:

(34) $$\left\{\begin{array}{c} z_{i}\geq \theta \Rightarrow \hat{c_{i}}=0\\ z_{i}\;<\theta \Rightarrow \hat{c_{i}}=1 \end{array}\right.$$

where $z_{i}$ denotes the score corresponding to the i-th observation, and $\hat{c_{i}}$ denotes the predicted class. For an established threshold, the classifier will be characterized by a specific TPR and a specific FPR; this pair represents a point in the FPR-TPR plane that describes the classifier. By varying the threshold, a new model will be obtained, characterized by different TPR and FPR corresponding to a new point in the plane. The set of points obtained by varying the threshold is called the ROC curve and represents a fundamental element for evaluating the classifier’s performance. The area under curve (AUC) indicates the capability of the classifier to separate the two classes.

#### 2.9.2. Feature Selection

To reduce dimensionality and avoid overfitting, we implemented the LASSO algorithm for feature selection. Starting from an observation $y_{i}$ characterized by a set of features and a class to be predicted, linear regression is based on the hypothesis that the observation can be obtained through a linear combination of the features plus an error term $\varepsilon_{i}$.

Let $x_{i}^{⊤}=\left(1,x_{i1},x_{i2},\ldots ,x_{ip}\right)$ be the row vector representing the features associated with the observation, and $\beta^{⊤}=\left(\beta_{0},\beta_{1},\beta_{2},\ldots ,\beta_{p}\right)$ the vector of coefficients weighting the features. Linear regression is defined as:

(35) $$y_{i}=\sum_{j=0}^{p}\beta_{j}x_{ij}+\varepsilon_{i}$$

where $\varepsilon_{i}$ represents the unknown error term. The prediction obtainable using a generic vector $\beta^{⊤}$ is:

(36) $$\hat{y_{i}}=\sum_{j=0}^{p}\beta_{j}x_{ij}$$

The prediction error is defined as the difference between the observed and predicted value. Linear regression aims to find the optimal vector $\hat{\beta }$ that minimizes the error over the n observations of the dataset:

(37) $$\hat{\beta }=\arg\underset{\beta }{\min}\sum_{i=1}^{n}{\left(y_{i}-\sum_{j=0}^{p}\beta_{j}x_{ij}\right)}^{2}$$

To perform feature selection, criteria, often statistical or based on the elements of β, are used to eliminate less informative features based on their contribution to the model in predictive terms. LASSO allows for feature selection by introducing a penalization term into the minimization problem:

(38) $$\hat{\beta }=\arg\underset{\beta }{\min}\sum_{i=1}^{n}{\left(y_{i}-\sum_{j=0}^{p}\beta_{j}x_{ij}\right)}^{2}+\lambda \sum_{j=0}^{p}\left|\beta_{j}\right|$$

where λ is the penalty parameter. If many coefficients have large values, the penalty term has a stronger impact. This procedure can shrink several coefficients to zero, facilitating the identification of features to remove. For binary classification tasks, logistic regression is used instead. In the linear approach, considering data generation, $y_{i}$ can be treated as a realization of a random variable $Y_{i}$, given a fixed error value. It can be shown that:

(39) $$E\left[Y_{i}|x_{i}\right]=\beta_{0}+\beta_{1}x_{i1}+\beta_{2}x_{i2}+\cdots +\beta_{p}x_{ip}$$

This expression identifies the deterministic part of the model. For dichotomous outcomes [37], this expected value corresponds to the conditional probability that $Y_{i}$ belongs to class 1:

(40) $$E\left[Y_{i}{|}_{x}\right]=P\left(Y_{i}=1|x_{i}\right)=\frac{1}{1+e^{-\left(\beta_{0}+\beta_{1}x_{i1}+\beta_{2}x_{i2}+\cdots +\beta_{p}x_{ip}\right)}}$$

Let $P(Y_{i}|x_{i})$ denote the probabilities that the model explains the true value of the observation; the final model is constructed from the product of probabilities for each observation:

(41) $$L\left(\beta \right)=\prod_{i=1}^{n}P\left(Y_{i}{|}_{x}\right)=\prod_{i=1}^{n}p_{i}^{Y_{i}}{\left(1-p_{i}\right)}^{1-Y_{i}},\;p_{i}=P\left(Y_{i}=1|x_{i}\right)$$

Taking the logarithm yields:

(42) $$l\left(\beta \right)=\log L\left(\beta \right)=\sum_{i=1}^{n}\left(Y_{i}\log p_{i}+\left(1-Y_{i}\right)\log\left(1-p_{i}\right)\right)$$

LASSO introduces a penalty term to this function as well:

(43) $$\hat{\beta }=\arg\underset{\beta }{\min}-\sum_{i=1}^{n}\left(Y_{i}\log p_{i}+\left(1-Y_{i}\right)\log\left(1-p_{i}\right)\right)+\lambda \sum_{j=0}^{p}\left|\beta_{j}\right|$$

#### 2.9.3. LDA Model Development

Let us consider the vector $x_{i}=\left[x_{i1},\;x_{i2},\;\ldots ,\;x_{id}\right]$, identifying the features associated with the i-th observation, and the vector $w=\left[w_{1},\;w_{2},\;\ldots ,\;w_{d}\right]$ representing a direction in the multidimensional space $R^{d}$. LDA consists of projecting the vector $x_{i}$ onto the direction identified by w, obtaining the score $z_{i}$ representative of the observation. To ensure that the classification is reliable, it is appropriate to identify the optimal vector w that guarantees maximum separability of the classes. The optimal vector corresponds to the direction that guarantees maximum separability between the centres of the distributions and minimum intra-class variance.

#### 2.9.4. SVM Model Development

The SVM algorithm identifies an optimal separating hyperplane that maximizes the minimum distance between the decision boundary and the training samples of each class. The separating hyperplane is defined as:

(44) $$w^{T}x+b=0$$

where w  denotes the normal vector to the hyperplane and b the bias term. The optimal hyperplane is obtained by solving the following convex optimization problem:

(45) $$\underset{w,b}{\min}\frac{1}{2}{\left\|w\right\|}^{2}$$

subject to:

(46) $$y_{i}\left(w^{T}x_{i}+b\right)\geq 1\;\;\forall i$$

where $y_{i}\in \{-1,+1\}$ are the class labels. Once the optimal hyperplane is estimated, a new sample x is classified according to:

(47) $$f\left(x\right)=\mathrm{sign}\left(w^{T}x+b\right)$$

## 3. Results

The results are presented according to the three main objectives: informative validation of the proposed features, robustness validation and evaluation of predictive modelling.

### 3.1. Correlation Results

**Figure 9:**
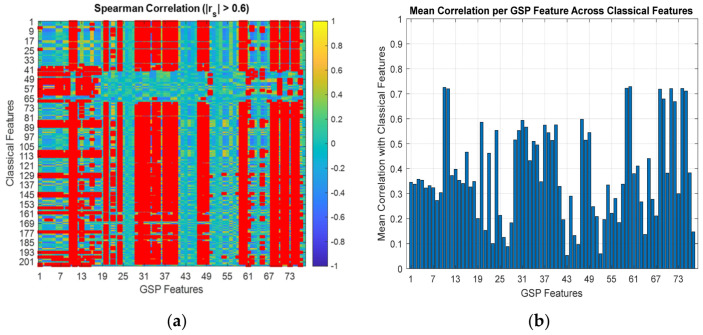
Spearman correlation: (**a**) Pairwise correlation matrix, where highly correlated features ($\left|r_{s}\right|\geq 0.6$) are highlighted by red rectangular markers; (**b**) Average correlation coefficient of each GSP feature against all conventional features, quantifying the overall feature overlap and redundancy.

The correlation analysis was performed setting a threshold of 0.6 to define a high correlation, in accordance with conventional interpretation guidelines [44,45]. Figure 9 reports the correlation matrix (a), where each element above the threshold is highlighted with a rectangular red marker, and the average correlation of each GSP feature with all classic features (b).

According to a Spearman correlation threshold of 0.6, 28.6% of GSP features were not correlated with any classical feature, indicating that these features provide additional information beyond the classical radiomics metrics. The median of the distribution in Figure 9b was compared with the reference values reported in Table 2, and the corresponding *p*-values were calculated.

### 3.2. Robustness Results

#### 3.2.1. Dependence on Segmentation

For each of the 10 lesions selected for the analysis, the mean, median, and standard deviation of the percentage difference vectors of the features were computed. Table 3 reports the results.

#### 3.2.2. Dependence on Batch Effects

In Table 4, the percentages of GSP and classical features for which the comparison yielded a q-value lower than the reference value α=0.05 are reported, respectively.

### 3.3. Predictive Modelling Results

#### 3.3.1. LDA Results

**Figure 10:**
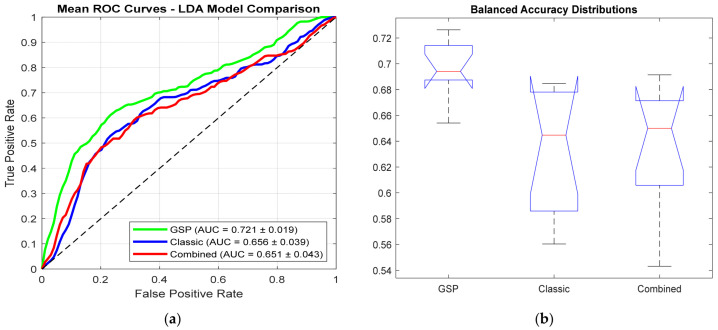
ROC analysis and Balanced Accuracy distributions (LDA): (**a**) Aggregated mean ROC curves for the classification models across all cross-validation runs; (**b**) Comparison of Balanced Accuracy distributions among the three distinct model configurations.

This section reports the results obtained by training the LDA classifier (see Section S2 in the Supplementary File for more details about the selected features and the most stable predictors). Figure 10 shows the mean ROC curves (a), obtained by averaging the curves across cross-validation runs, and the distributions of balanced accuracies for the three models (b).

Table 5 reports the performance metrics for the three models, including Accuracy, AUC, Precision, Recall, Specificity, F1-score, and Balanced Accuracy. All metrics are reported as mean values ± half-width of the corresponding 95% confidence intervals.

**Figure 11:**
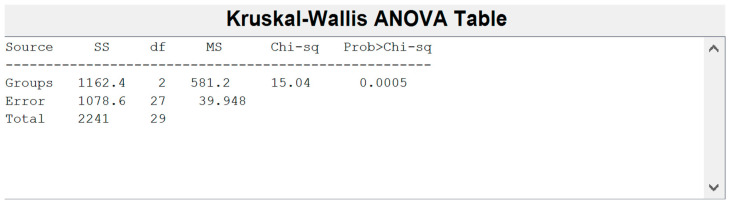
Kruskal–Wallis test result (LDA).

From an inferential perspective, the distributions of balanced accuracies across the different runs were tested using a Kruskal–Wallis test. The result is shown in Figure 11.

From this plot, it was possible to identify that at least one group differed significantly from the others. A Dunn–Šidák post hoc test [46,47] was then applied to verify if the GSP model performed better than the others. The results are reported in Table 6.

#### 3.3.2. SVM Results

**Figure 12:**
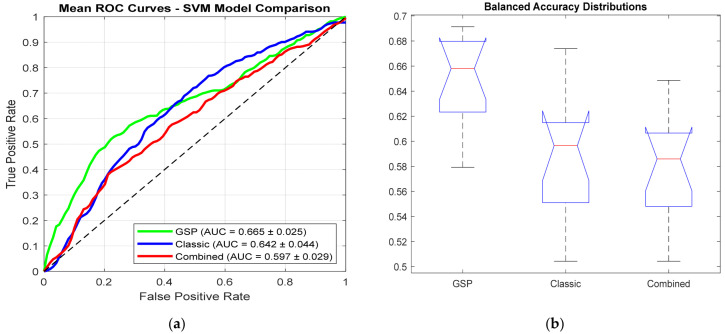
ROC analysis and Balanced Accuracy distributions (SVM): (**a**) Aggregated mean ROC curves for the classification models across all cross-validation runs; (**b**) Comparison of Balanced Accuracy distributions among the three distinct model configurations.

This section reports the results obtained by training the SVM classifier. Figure 12 shows the mean ROC curves (a), obtained by averaging the curves across cross-validation runs, and the distributions of balanced accuracies for the three models (b).

Table 7 reports the performance metrics for the three models, including Accuracy, AUC, Precision, Recall, Specificity, F1-score, and Balanced Accuracy. All metrics are reported as mean values ± half-width of the corresponding 95% confidence intervals.

**Figure 13:**
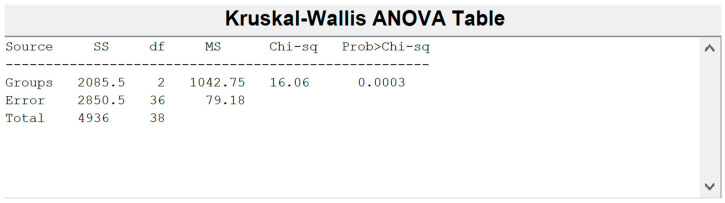
Kruskal–Wallis test result (SVM).

From an inferential perspective, the distributions of balanced accuracies across the different runs were tested using a Kruskal–Wallis test. The result is shown in Figure 13.

From this plot, it was possible to identify that at least one group differed significantly from the others. A Dunn–Šidák post hoc test was then applied to verify if the GSP model performed better than the others. The results are reported in Table 8.

## 4. Discussion

This study presents an exploratory investigation of a novel graph-based radiomics methodology [48,49]. The results of the correlation study suggest that the proposed features capture discriminative patterns not entirely encoded by standard voxel-based descriptors, while the classification results suggest that the proposed features provide additional predictive information. The proposed graph-based metrics aim to describe the geometry, structure, and characteristics of the metabolic activity of the lesion. The orientation features highlight important surface characteristics of the lesion, such as general elongation, or possible anisotropies in different directions, by examining the distribution of the normals to the triangles composing the mesh. From a biological perspective, a high angular entropy or isotropic distribution of mesh surface normals may indicate a disorganized, infiltrative growth pattern typical of aggressive malignant processes, where tumour cells disrupt the regular bone architecture symmetrically in multiple directions. Conversely, lower entropy and dominant directional patterns might map localized, slow-growing benign remodelling that follows predictable anatomical stress lines. The connectivity metrics, instead, highlight the overall structure of the lesion, investigating the density and inhomogeneity of internal and external connections, to infer the possible presence of regular, symmetric regions and to determine how globally cohesive the lesion is, consistently with the etiopathological framework of the target. A highly dense and inhomogeneous internal connection network reflects a fractured, multi-focal biological structure, which aligns with the disorganized tissue layout seen in osteoblastic or mixed metastatic lesions. Metrics derived from transforms highlight complementary characteristics of the metabolic signal associated with the target. The dual-band energy partitioning via the GFT serves as a digital surrogate for metabolic clustering: high-frequency spectral energy maps localized fluctuations and sharp metabolic peaks, potentially corresponding biologically to hypermetabolic angiogenic hotspots or highly proliferative tumour nests. On the other hand, multi-scale GWT coefficients track how radiotracer uptake transitions from localized microscale variations to macroscale patterns. This multi-resolution filtering allows the identification of distinct metabolic sub-regions (tumour habitats), reflecting the intrinsic intra-lesion functional heterogeneity that drives prostate cancer malignancy and therapeutic resistance. Transform-derived features may be particularly useful for describing complex metabolic activity patterns, thereby providing a useful tool for identifying and differentiating lesions at different stages of disease progression. These aspects may be particularly relevant for distinguishing benign from malignant lesions, as they reflect differences in structural organization and metabolic heterogeneity. The interpretability of the metrics is therefore closely linked to the surface, structural, and metabolic aspects of the lesion. Although these biological interpretations remain hypothetical, they provide a plausible explanation for the observed discriminative performance of the proposed graph-based descriptors and motivate their further investigation in larger validation cohorts.

In particular, the models trained exclusively on GSP features exhibited higher balanced accuracy and AUC than the other two models. These findings suggest that the information captured through the proposed representation may improve class separation and intrinsic robustness to class imbalance compared with classical radiomics features, while providing no evident benefit when combined with the standard feature domain. We have verified that the GSP metrics exhibit greater robustness to variability introduced by segmentation and to batch effects; therefore, the results obtained may reflect these improvements. Despite these encouraging findings, we fully acknowledge that this study is limited by its single-centre design, a relatively small and unbalanced cohort, and the lack of an independent external validation cohort. These represent intrinsic limitations, particularly for machine learning-based approaches where model generalizability can be highly sensitive to centre-specific imaging protocols and patient demographics. Although the proposed framework demonstrated robustness against scanner-related batch effects and segmentation variability within the available dataset, future prospective, multicentre studies incorporating diverse external cohorts are strictly necessary to further evaluate and validate the generalizability and clinical applicability of the proposed graph-based radiomics approach. In particular, future validation studies will be designed to include heterogeneous multicentre cohorts acquired with different scanners, acquisition protocols, and patient populations, allowing the assessment of the generalizability and reproducibility of the proposed graph-based radiomics features across diverse clinical environments. This multicentre evaluation will also enable the assessment of model calibration, stability, and potential centre-specific performance variability, providing a necessary step toward future clinical translation. Furthermore, the evaluation does not include direct comparisons with more recent or advanced approaches, such as deep learning–based methods or state-of-the-art radiomics approaches. This choice was primarily driven by the limited size of the available dataset, which we believe would not support a fair, stable, and meaningful training and evaluation of more complex models without a high risk of overfitting. For these reasons, we emphasize that the results should be interpreted as preliminary and hypothesis-generating rather than definitive. Future studies will investigate the discriminative capabilities of the models on larger datasets, incorporating complementary techniques to classical GSP, such as Graph Neural Networks (GNN) [50,51] and Topological Signal Processing (TSP) [27,52]. These approaches will be introduced to provide complementary information beyond that captured by the proposed features, with the goal of maximizing their discriminative power within this translational framework. These investigations will be performed within larger multicentre datasets, enabling independent external validation of the proposed framework and a more comprehensive assessment of its clinical generalizability and robustness across different institutions.

## 5. Conclusions

This study presents an exploratory translational framework for integrating graph-based signal processing into radiomics analysis. The proposed methodology demonstrated the potential to capture complementary morpho-functional characteristics that are not directly represented by conventional voxel-based radiomics features, suggesting a potential improvement in predictive performance. Although the findings remain preliminary, they support the feasibility of the proposed approach and motivate further investigation. Future studies will focus on larger multicentre cohorts and the integration of advanced graph-based processing strategies to externally validate the proposed framework and further assess its robustness, generalizability, and potential clinical applicability.

## Figures and Tables

**Table 1:** EWMA filter parameters.

EWMA Filter Parameters
$\alpha_{min}\;=0.2$
$\alpha_{max}=0.9$
σ=0.25

**Table 2:** Wilcoxon test results. Baseline reference values and corresponding *p*-values derived from the statistical comparison against the GSP feature correlation distribution shown in Figure 9b.

Reference	*p*-Value
0.5	0.0001
0.45	0.0027
0.4	0.22

**Table 3:** Dependence on segmentation. Mean, median, and standard deviation of the percentage difference vectors calculated across the 10 selected lesions for both GSP and classical features.

Lesion	Features	Mean Perc. Diff.	Median Perc. Diff.	S.D. Perc. Diff.
L1	GSP	−0.97%	2.09%	116.85%
L1	Classical	53.38%	7.92%	156.15%
L2	GSP	1.3%	−0.23%	54.05%
L2	Classical	8.61%	−2.36%	173.2%
L3	GSP	26.49%	0.81%	173.2%
L3	Classical	123.41%	1.34%	1244.53%
L4	GSP	8.84%	−3.37%	131.14%
L4	Classical	12.05%	−5.3%	157.28%
L5	GSP	−19.79%	−3.79%	108.14%
L5	Classical	66.92%	−7.52%	923.12%
L6	GSP	−4.43%	−3.91%	36.5%
L6	Classical	−27.2%	−4.92%	120.33%
L7	GSP	−2.42%	0.18%	11.48%
L7	Classical	3.62%	1.34%	53.36%
L8	GSP	1.83%	1.13%	17.33%
L8	Classical	4.5%	1.16%	28.04%
L9	GSP	−5.95%	−2.15%	59.05%
L9	Classical	50.83%	−4.72%	762.13%
L10	GSP	−3.9%	−1.66%	11.15%
L10	Classical	−5.62%	−1.7%	95.54%

**Table 4:** Dependence on batch effects. Percentage of GSP and conventional radiomics features satisfying the significance criteria.

Features	Percentage Affected by Batch Effects
GSP	59.7%
Classical	76.2%

**Table 5:** LDA performance metrics expressed as mean ± 95% confidence interval half-width.

Model	Accuracy	AUC	Precision	Recall	Specificity	F1-Score	B. Accuracy
GSP	0.7364 ± 0.019	0.7214 ± 0.0187	0.3874 ± 0.0236	0.6176 ± 0.0358	0.776 ± 0.0261	0.4746 ± 0.0188	0.6968 ± 0.0149
Classic	0.7222 ± 0.025	0.6555 ± 0.0386	0.344 ± 0.0397	0.4824 ± 0.0621	0.7907 ± 0.0246	0.4002 ± 0.0459	0.6365 ± 0.0325
Combined	0.7265 ± 0.0307	0.6511 ± 0.0429	0.3537 ± 0.0499	0.4706 ± 0.0444	0.8 ± 0.0302	0.4027 ± 0.0469	0.6353 ± 0.0338

**Table 6:** Post hoc comparison results (LDA).

Model 1	Model 2	Rank Difference	*p*-Value
GSP	Classic	22.79	0.002
GSP	Combined	22.39	0.0028
Classic	Combined	8.99	0.99

**Table 7:** SVM performance metrics expressed as mean ± 95% confidence interval half-width.

Model	Accuracy	AUC	Precision	Recall	Specificity	F1-Score	B. Accuracy
GSP	0.7411 ± 0.0195	0.6649 ± 0.0248	0.3615 ± 0.0306	0.5113 ± 0.0337	0.7918 ± 0.0234	0.422 ± 0.0286	0.6516 ± 0.0207
Classic	0.6832 ± 0.0278	0.6419 ± 0.0438	0.2898 ± 0.0321	0.4072 ± 0.0532	0.7713 ± 0.0294	0.3364 ± 0.0368	0.5893 ± 0.0273
Combined	0.6843 ± 0.0194	0.5974 ± 0.0287	0.2734 ± 0.0324	0.3982 ± 0.0439	0.7579 ± 0.0225	0.3233 ± 0.0356	0.5781 ± 0.0264

**Table 8:** Post hoc comparison results (SVM).

Model 1	Model 2	Rank Difference	*p*-Value
GSP	Classic	24.71	0.0051
GSP	Combined	27.33	0.0006
Classic	Combined	13.29	0.91

## Data Availability

The data presented in this study are available on request due to institutional restrictions from the corresponding author.

## Supplementary Materials

The following supporting information can be downloaded at: https://www.mdpi.com/article/10.3390/diagnostics16142284/s1. Section S1: Pointcloud density; Section S2: Selected Features.
