# Supplementary material for: Enhancing PET/CT Radiomics Robustness Through Graph Signal Processing
Source: Diagnostics (Basel). 2026 Jul 21;16(14):2284. doi: 10.3390/diagnostics16142284 (PMC13408087; doi:10.3390/diagnostics16142284)
Supplement: Supplementary file 1 [file diagnostics-16-02284-s001.zip › diagnostics-4372570-supplementary.pdf]

In this file, we report the additional analyses and methodological details which have not been included in the main manuscript.

Section S1: Pointcloud density

The number of points used allows us to obtain a balance between the goodness of the geometric representation and computational times. To verify this aspect, 10 of the 92 lesions were selected, and for each lesion three different point clouds were extracted, respectively with 300, 600 and 800 points. For each point cloud, the mesh was constructed and the GSP features were extracted, monitoring the total analysis times. To verify how the metrics varied among the representations, the feature vector related to the 300-point cloud was compared element by element with the vector related to the 600-point cloud, and the distribution of the percentage variation with respect to the vector related to the denser point cloud was constructed. The mean, median and standard deviation of this distribution were calculated. The same approach was repeated to compare the vectors related respectively to the 600- and 800-point clouds. The results are reported in Table s.1, while in Table s.2 the average computational times required to carry out the meshes and the GSP analysis for each point cloud density are reported.

Lesion	PC 1 Density	PC 2 Density	Mean Perc.diff.	Median Perc. diff.	S.D. Perc. Diff.
L1	300	600	-75.27 %	-7.35 %	275.71 %
L1	600	800	5.97 %	1.93 %	204.19 %
L2	300	600	-20.63 %	-1.25 %	236.3 %
L2	600	800	16.36 %	-0.43 %	70.27 %
L3	300	600	-187.08 %	-3.02 %	961.23 %
L3	600	800	8.33 %	1.45 %	84.12 %
L4	300	600	-73.74 %	1.87 %	760.4 %
L4	600	800	28.68 %	-0.43 %	220.33 %
L5	300	600	-20.05 %	-4.21 %	575.79 %
L5	600	800	-6.58 %	1.66 %	57.66%
L6	300	600	-15.9 %	1.75 %	191.32 %
L6	600	800	-8.07 %	-0.89 %	133.67 %
L7	300	600	-108.82 %	-3.38 %	623.22 %
L7	600	800	29.09 %	-0.48 %	164.96 %
L8	300	600	37.41 %	-1.97 %	345.8 %
L8	600	800	-2.24 %	-0.67 %	145.78 %
L9	300	600	-12.41 %	-1.74 %	52.11%
L9	600	800	-1.5 %	-0.62 %	22 %
L10	300	600	-16.73 %	-3.93 %	62.68 %
L10	600	800	3.19 %	-1.7 %	51.05 %

Table S1. Feature variability evaluation

PC Density	Computational Times
300	35 seconds
600	3.27 minutes
800	8.45 minutes

Table S2. Average computational times

Considering the first comparisons, it can be inferred that, in general, most of the features do not vary significantly between the representations, as the medians are relatively low; however, by examining the mean and the standard deviation, it is possible to conclude that there exists a relatively large subset of metrics that vary substantially and are closely dependent on the density of the point cloud. In the comparisons between 300 and 600 points, both the means and the standard deviations are higher, indicating a greater variability. In contrast, for the comparisons between 600 and 800 points, the means and standard deviations are lower, showing a more contained and stable variation. The density of the point cloud therefore confirms itself as a crucial parameter that greatly influences the metrics. Dense representations undoubtedly provide a benefit in terms of reliability, since the connection with the underlying geometric structure is stronger; in this scenario, relying on point clouds that are too sparse entails the risk of extracting metrics that are very different and less representative of those obtained with denser representations. However, beyond a certain threshold, excessively increasing the density of the representation results in an exponential increase in analysis time without providing excessively significant changes to the metrics that would be extracted from less dense point clouds, characterized by a much lower analysis time. In this scenario, we chose 600 points as a trade-off between metric reliability and computational cost.

Section S2: Selected Features

For each model, the most stable features were identified by analyzing their selection frequency across the entire training procedure. Features selected in more than 50% of the 50 cross-validation feature-selection procedures were retained as the most stable predictors. Table s.3 reports the results.

Model	Feature	Frequency
GSP	IQR wavelet s = 100	100 %
GSP	median of the z-coordinate of the surface normals	89.39 %
GSP	90th percentile of the x-coordinate of the surface normals	68.18 %
GSP	90th percentile of the z-coordinate of the surface normals	60.61 %
Classic	Coarseness3D	74.24 %
Classic	SmallZoneLowGrayLevelEmphasis3D	68.18 %
Combined	IQR wavelet s = 100	100 %

Table S3. Stability of selected features across cross-validation procedures

The feature selection analysis highlights a strong stability of multiscale wavelet-based descriptors across all models, with the IQR of the wavelet coefficient at scale s = 100 being consistently selected in 100% of the cross-validation procedures. Since wavelet-derived features capture local variations in the metabolic signal, this result suggests that localized metabolic heterogeneity plays a dominant role in the predictive framework. GSP-derived geometric features also demonstrate high selection stability, particularly those related to the distribution of surface normal components, suggesting a strong relevance of lesion morphology and orientation. In contrast, classical radiomic texture features exhibit lower but still consistent selection frequencies, indicating a complementary but less dominant contribution to the predictive models.
